# Subversion of a family of antimicrobial proteins by *Salmonella enterica*

**DOI:** 10.3389/fcimb.2024.1375887

**Published:** 2024-03-05

**Authors:** Roman G. Gerlach, Irene Wittmann, Lena Heinrich, Olaf Pinkenburg, Torben Meyer, Laura Elpers, Christiane Schmidt, Michael Hensel, Markus Schnare

**Affiliations:** ^1^ Institute of Clinical Microbiology, Immunology and Hygiene, University Hospital of Erlangen and Friedrich-Alexander-University (FAU) Erlangen-Nuremberg, Erlangen, Germany; ^2^ Robert Koch Institute, Wernigerode, Germany; ^3^ Institute for Immunology, Philipps-University Marburg, Marburg, Germany; ^4^ Division of Microbiology and CellNanOs – Center of Cellular Nanoanalytics Osnabrück, School of Biology/Chemistry, University Osnabrück, Osnabrück, Germany

**Keywords:** antimicrobial proteins and peptides, type 1 fimbriae, *Salmonella* Typhimurium, adhesion, antimicrobial resistance, bactericidal permeability increasing protein

## Abstract

*Salmonella enterica* is a food-borne pathogen able to cause a wide spectrum of diseases ranging from mild gastroenteritis to systemic infections. During almost all stages of the infection process *Salmonella* is likely to be exposed to a wide variety of host-derived antimicrobial peptides (AMPs). AMPs are important components of the innate immune response which integrate within the bacterial membrane, thus forming pores which lead ultimately to bacterial killing. In contrast to other AMPs Bactericidal/Permeability-increasing Protein (BPI) displayed only weak bacteriostatic or bactericidal effects towards *Salmonella enterica* sv. Typhimurium (STM) cultures. Surprisingly, we found that sub-antimicrobial concentrations of BPI fold-containing (BPIF) superfamily members mediated adhesion of STM depending on pre-formed type 1 fimbriae. BPIF proteins directly bind to type 1 fimbriae through mannose-containing oligosaccharide modifications. Fimbriae decorated with BPIF proteins exhibit extended binding specificity, allowing for bacterial adhesion on a greater variety of abiotic and biotic surfaces likely promoting host colonization. Further, fimbriae significantly contributed to the resistance against BPI, probably through sequestration of the AMP before membrane interaction. In conclusion, functional subversion of innate immune proteins of the BPIF family through binding to fimbriae promotes *Salmonella* virulence by survival of host defense and promotion of host colonization.

## Introduction

Antimicrobial peptides and proteins (AMPs) are key players of the innate immune response against bacterial infections. One of the most multifaceted AMPs known today is Bactericidal/Permeability-increasing Protein (BPI). The C-terminal portion of BPI was shown to opsonize bacteria whereas the N-terminus can act anti-angiogenetic, neutralizes LPS via direct binding to Lipid A and harbors the antimicrobial activity. Bactericidal activity against Gram negative bacteria could be narrowed down to a 27 amino acids (aa) peptide, including residues 82 to 108 of human BPI (Aichele et al., 2006). HuBPI binds to the bacterial outer membrane (OM) resulting in membrane rupture, change of transmembrane potential and increase in membrane current (Wiese et al., 1997). A mouse ortholog of BPI (mBPI) was identified (Lennartsson et al., 2005) and LPS-neutralizing activity of mBPI was demonstrated (Wittmann et al., 2008). Several other proteins share a similar 3-dimensional structure with BPI and are referred to as the BPI-fold (BPIF)-family. The most prominent BPIF family member is lipopolysaccharide-binding protein (LBP). LBP binds LPS at the same moieties as BPI, although with a lower affinity and is mainly thought to be involved into the recognition of LPS and other bacterial cell envelope components by the immune system to initiate an immune response (Eckert et al., 2013). Recently, the palate, lung, and nasal epithelium clone associated proteins (PLUNC) were added to the BPIF superfamily. The PLUNC-subfamily can be divided into short (S) PLUNC proteins, which share homology to the N-terminal part of BPI and long (L) PLUNC-proteins, which are homologous to the complete BPI protein (Holweg et al., 2011). Finally, two lipid transfer proteins, phospholipid transfer protein (PLTP) and cholesteryl ester transfer protein (CETP), belong to the BPIF-family. These proteins have their main function outside the principle immune response (Elsbach and Weiss, 1998).

Binding and activity of antimicrobial peptides and proteins can be sensed by bacteria through specialized signaling complexes within the bacterial membrane. The signal transduction works through two component signal transduction (TCS) or phosphorelay systems which involves the activation of a membrane-bound sensor kinase and final phosphorylation of a response regulator which acts as a transcriptional activator (Otto, 2009). So far, the ApsSR system of *Staphylococcus epidermidis* (Li et al., 2007), the CovRS (CsrRS) system of *Streptococcus pyogenes* (Gryllos et al., 2008) and PhoPQ (Bader et al., 2005; Richards et al., 2012), PmrAB (Richards et al., 2012), as well as RcsCDF (Farris et al., 2010) of *Salmonella enterica* sv. Typhimurium (*S*. Typhimurium, STM) were identified to be directly involved in sensing of AMPs. Binding and integration of AMPs within the bacterial membrane results in activation of the extracytoplasmic stress response (Crouch et al., 2005). Besides the alternative sigma factor σ^E^ and the RcsCDF phosphorelay, the CpxRA TCS was shown to be required for this adaptation in *E. coli* (Audrain et al., 2013). Although the systems differ in their downstream mechanisms, activation results generally in higher resistance against AMPs. Resistance is achieved by membrane modifications, lowering the binding affinity of AMPs, active export or proteolytic inactivation of AMPs (Peschel and Sahl, 2006). Results for *Streptococcus* indicate that virulence functions can be induced in response to AMP binding (Gryllos et al., 2008). Furthermore, PhoPQ and CpxRA of STM are involved in regulating bacterial virulence (Groisman, 2001; Humphreys et al., 2004).

*Salmonella enterica* colonizes a broad range of different hosts and is one of the most prevalent causes of bacterial enteritis in humans. One reason of this broad host range is thought to be the vast set of adhesive surface structures encoded within their genomes. Sequencing of STM revealed the presence of at least five non-fimbrial adhesins and 13 fimbrial operons (McClelland et al., 2001; Wagner and Hensel, 2011). Type 1 fimbriae are the only fimbriae expressed under standard laboratory conditions and were shown to bind to mannose residues (Humphries et al., 2003). Adhesion to some epithelial cell lines depends on functional type 1 fimbriae (Bäumler et al., 1996) but in a mouse model of infection, alternative fimbrial structures can compensate for deficiency of type 1 fimbriae (van der Velden et al., 1998). This is in line with expression demonstrated for 9 of the 13 different fimbriae encoded by *S*. Typhimurium *in vivo* (Humphries et al., 2003). The environmental cues responsible for the induction of fimbriae expression in the intestine still need to be defined.

Type 1 fimbriae biogenesis follows the chaperone usher pathway, which was elucidated in great detail for *E. coli* (Capitani et al., 2006). FimD forms an outer membrane assembly platform, the usher. Individual subunits are transported in a Sec-dependent manner into the periplasmic space where they bind to cognate chaperones. The chaperone subunit complex is recognized by the usher which transports the fimbrial subunit to the bacterial surface thus polymerizing the pilus (Capitani et al., 2006). Polymerization is very stable and works by a strand complementation mechanism where an adjacent subunit completes a protein fold by donating a β-strand (Puorger et al., 2008). The pilus is assembled in a top-to-bottom order and for *E. coli* the requirement of the tip adhesin FimH for pilus biogenesis was experimentally shown (Munera et al., 2007). Adhesion mediated by surface structures like fimbriae is required for successful intestinal colonization of *S.* Typhimurium enabling subsequent steps of pathogenesis (Humphries et al., 2003).

We aimed to characterize the effect of BPI and other BPIF family-members on STM. Surprisingly, BPI exerted only a weak antibacterial activity against STM but at the same time stimulated *Salmonella* adhesion. By applying a comprehensive series of analyses, we found that BPIF family AMPs can trigger adhesion of *Salmonella* via direct interaction with type 1 fimbriae. Type 1 fimbriae also contributed significantly to the observed resistance against BPI, probably by preventing further interaction of the AMP with the bacterial OM. Here we identified a mechanism where antimicrobial host activities were not only neutralized, but rather used to promote virulence functions (adhesion) of the pathogen. Our findings exemplify that a previously characterized virulence factor with defined molecular interactions can exhibit additional functions in the complex setting of host immune defenses.

## Results

### BPI has only moderate bactericidal effects on *Salmonella* but can bind to the bacterial surface

**Figure 1:**
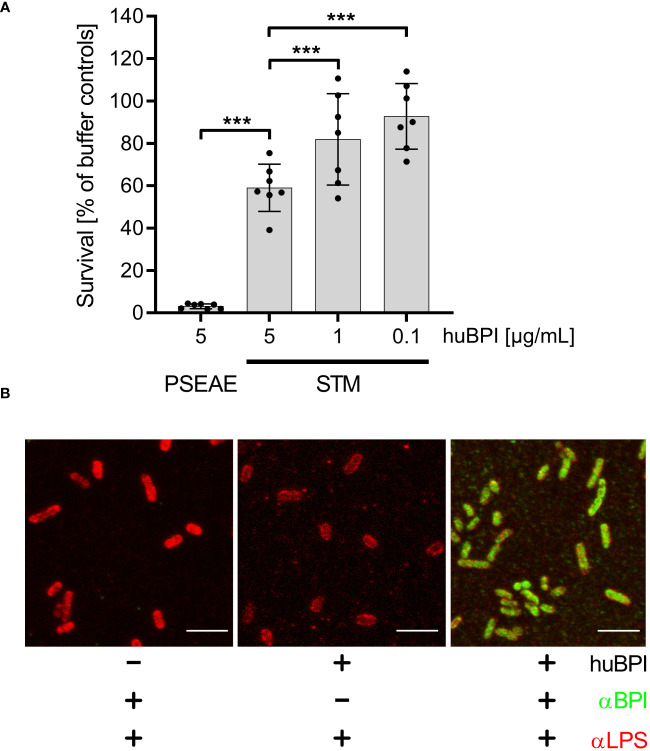
Impact of huBPI on bacterial survival and binding of huBPI to the *Salmonella* cell envelope. **(A)** Bacterial survival after one hour incubation with the indicated concentrations of huBPI was quantified by plating. *P. aeruginosa* (PSEAE) and of *S*. Typhimurium (STM). The results are presented as mean ± SD of 3 to 4 independent experiments. Statistical significance was calculated using one-way ANOVA and was defined as *** for adj. *p* < 0.001. **(B)** STM was incubated for 2 h with 1 µg/mL huBPI (“+”) or with buffer control (“-”) and subsequently fixed on glass coverslips. STM were labeled using an anti-LPS (αLPS, red) and anti-huBPI was used to detect huBPI (αBPI, green). STM were detected by confocal laser-scanning microscopy. Scale bars = 5 µm.

The antimicrobial protein BPI has been shown to have a strong bactericidal effect on mucoid and multiresistant *Pseudomonas aeruginosa* strains in a time- and concentration-dependent manner (Aichele et al., 2006). Incubation of *P. aeruginosa* ATCC 27853 with 5 µg/mL BPI purified from human neutrophils (huBPI) for 60 minutes reduced colony forming units (CFU) by ~95% in a microtiter-plate assay (**Figure 1A**). When *S*. Typhimurium was exposed to huBPI in concentrations of 0.1, 1 and 5 µg/mL, only moderate bactericidal effects were observed, resulting in survival of 95%, 81% and 67% of the bacteria compared to controls, respectively (**Figure 1A**).

BPI showed varying antimicrobial activity against different strains of STM (Weiss et al., 1978). Since we observed only a weak antimicrobial activity of BPI against *S*. Typhimurium NCTC 12023, we analyzed whether BPI could associate with the surface of the strain. Therefore, we incubated STM with the AMP and detected the binding using a monoclonal antibody directed against huBPI. Confocal microscopy verified binding of huBPI to the bacterial surface and co-staining with an anti-LPS antibody revealed a complete colocalization of huBPI to the bacterial LPS (**Figure 1B**). Furthermore, we observed weaker LPS staining in the presence of huBPI, which might be indicative that BPI interferes with LPS antigen accessibility (**Figure 1B**).

### BPIF family members promote adhesion of *Salmonella* Typhimurium

**Figure 2:**
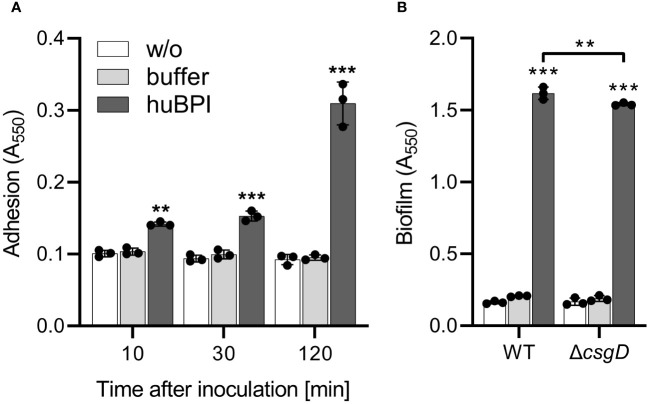
BPI promotes adhesion to plastic surfaces independent of its capacity to form biofilms. **(A)** Binding of *S*. Typhimurium WT to plastic surfaces was quantified at the time points indicated after incubation in LB with addition of 1 µg/mL huBPI (dark grey bar), BPI buffer (grey bar) or LB medium without bacteria (w/o, white bar). **(B)** Assay as described in **(A)** but carried out under biofilm-forming conditions (LB without salt, 28°C) for 24h Bound bacteria were quantified using crystal violet. The results are presented as mean ± SD of 3 to 4 independent experiments. Statistical significance was calculated between controls (w/o, buffer) and treated or between groups as indicated using one-way ANOVA defined as ** for adj. *p* < 0.01 and *** for adj. *p* < 0.001.

As shown above, huBPI readily bound to the surface of STM cells but had only a minor effect on the bacterial viability. Because surface-localized adhesive structures are key virulence factors of *Salmonella* (Gerlach and Hensel, 2007), we analyzed whether surface-binding of huBPI would interfere with the adhesion properties of *Salmonella*. Bacterial adhesion was assessed in a 96-well plate by staining the bound bacterial cells with crystal violet (CV) (Kai-Larsen et al., 2010). To our surprise, *Salmonella* started to adhere to the well surface shortly after addition of a non-bactericidal concentration of huBPI (1 µg/mL). Addition of huBPI led to an increase of CV staining over time indicating an accumulation of bacteria at the well surface (**Figure 2A**).

In order to determine whether the adhesion-inducing activity of BPI could also be seen in other BPIF family members, we recombinantly expressed short palate, lung, and nasal epithelium clone protein 1 (SPLUNC1 = BPIFA), long PLUNC 1 = BPIFB), as well as LBP in insect cells. All recombinant proteins carried a His epitope tag, which allowed for detection and purification. The purity of the recombinant proteins was estimated over 90% (data not shown). All of the tested BPIF proteins induced binding of STM to plastic surfaces (**Supplementary Figures 1A, B**). To exclude an unspecific effect of the expression or purification system on bacterial adhesion, we expressed and purified a mouse procryptdin protein by the same means. As expected, the mouse procryptdin protein did not influence the adhesion of the bacteria (**Supplementary Figure 1C**).

BPI, like many other AMPs, displays its antibacterial activity due to its cationic nature. Given the similar mode of action we were wondering whether other antimicrobial peptides would also induce adhesion of STM. Therefore, we tested human α-defensin 5 (HD5, as representative of α-defensins), human β-defensin 2 (HBD2, a representative of β-defensins) and LL-37 (cathelicidin). Although all three peptides showed their bactericidal activity when tested against *P. aeruginosa*, none of the peptides enhanced adhesion in *Salmonella* (data not shown).

In a next step, we tested whether huBPI could promote *Salmonella* biofilm formation because (1) biofilms are often observed on abiotic surfaces and (2) adhesion is required as an initial step of the biofilm formation process. *S*. Typhimurium can form biofilms *in vitro* under low temperature (28°C) and low osmolarity (medium without salt) (Römling et al., 1998). With the addition of 1 µg/mL huBPI, we observed a strong surface binding of bacteria after 24 h incubation under biofilm-promoting conditions (**Supplementary Figure 1D**). Furthermore, higher concentrations of HD5 as well as HBD2 inhibited biofilm formation of *S*. Typhimurium most likely via their antimicrobial action at this concentration. Whereas LL-37 had no detectable impact on *Salmonella* biofilm formation (**Supplementary Figure 1D**).

Because CV-based bacterial quantification is rather unspecific and cannot discriminate between bacterial adhesion and biofilm formation, an isogenic *Salmonella* mutant strain deficient for *csgD* was generated. The transcription factor CsgD is essential for the expression of the biofilm matrix components curli and cellulose (Römling et al., 1998). As expected, the *csgD* mutant did not produce a biofilm when grown on plates under biofilm-inducing conditions (data not shown). However, with addition of huBPI, STM Δ*csgD* started to adhere to the cell culture plate to almost the same extent as WT (**Figure 2B**). Similar results were obtained using *Salmonella* mutants with defects in curli or cellulose structural genes (data not shown) speaking against a significant involvement of biofilm formation in the observed phenotype.

### BPIF protein-induced adhesion is a post-translational process

**Figure 3:**
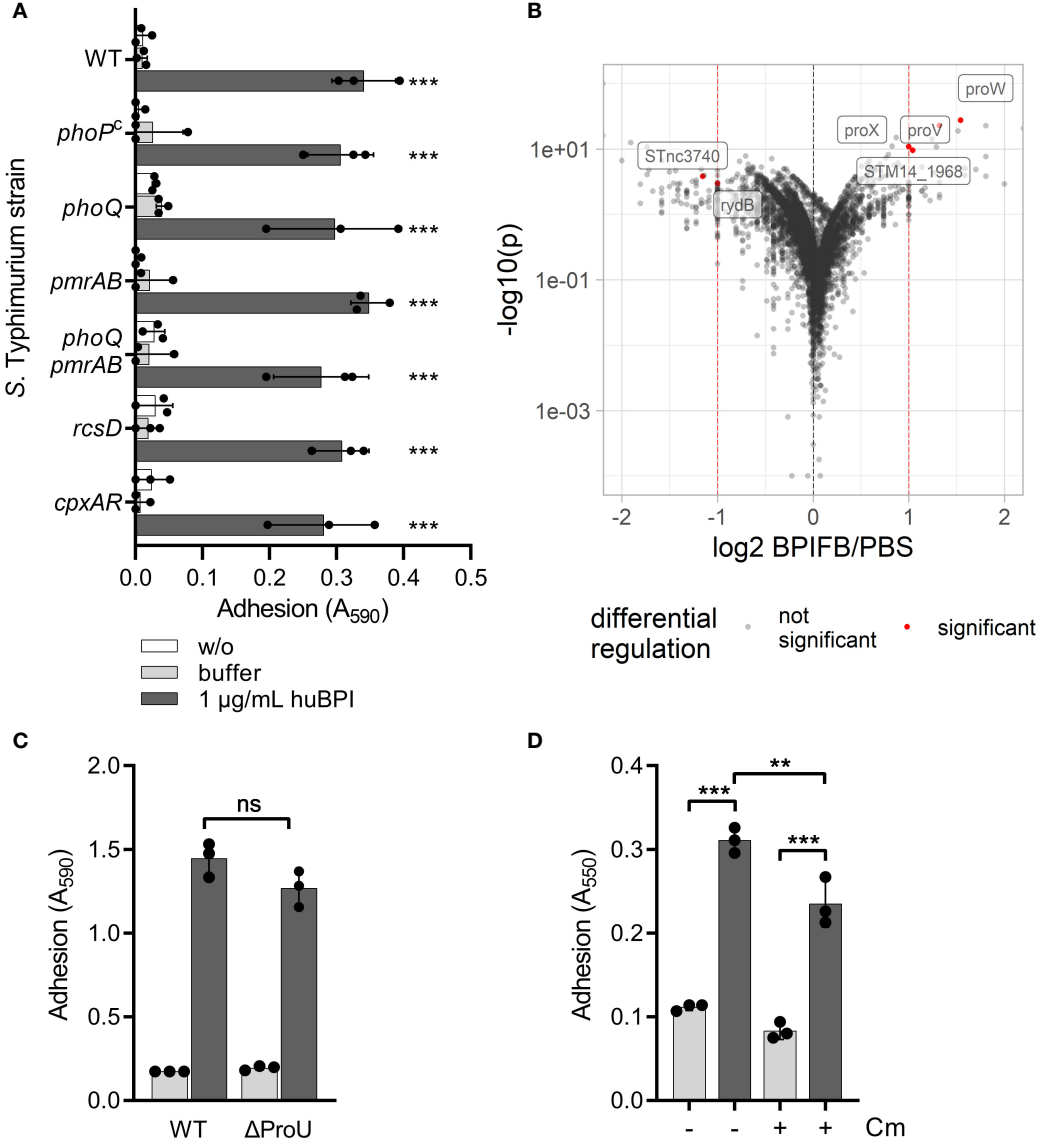
BPIF-induced adhesion is a post-translational process. **(A)** A crystal violet (CV) assay was used to quantify binding of different isogenic *S*. Typhimurium mutants after 2h **(B)** Dot plot of RNA-seq results. Each dot corresponds to a gene for which RNA-seq data was obtained (n = 5,055). Log2 of relative expression of BPIFB (LPLUNC1)-treated bacteria vs. PBS controls was plotted against -log10 of the *p* value. Red dashed lines show the thresholds used for relative expression (>2-fold). Labels and red dots represent significantly regulated genes. **(C)** Adhesion of ΔProU (Δ*proVWX*) to plastic surface after treatment with huBPI (BPI; dark grey bars) or buffer (light grey bars) for 4 h was quantified by crystal violet staining. **(D)** Crystal violet assay after 2 h with or without block of protein *de novo* synthesis using chloramphenicol (Cm) where indicated. The results shown are presented as mean ± SD of three independent experiments done in triplicates. Statistical significance was calculated using one-way ANOVA **(A, D)** or a two-tailed unpaired Student’s *t* test **(C)** and was defined as ** for *p* < 0.01 and *** for *p* < 0.001, ns, not significant.

We speculated that signal transduction from one or more of the known AMP-detecting signaling complexes of STM might be involved in BPIF protein-induced adhesion. To address this, isogenic mutants deficient for *phoQ*, *pmrAB*, *rcsD* and *cpxRA* or with constitutively active PhoPQ system (PhoP^c^) (Gunn et al., 1996) were tested. To exclude overlapping functions of the PhoPQ and PmrAB systems (Richards et al., 2012; Johnson et al., 2013), a mutant deficient for both TCSs was generated. None of these STM mutants showed a defect in the huBPI-induced adhesion (**Figure 3A**). Therefore, we concluded that huBPI induces adhesion of STM independent of known signal transduction systems involved in AMP sensing.

Next, we did differential transcriptome analysis using RNA-seq to elucidate the transcriptional changes which might be induced by BPIF family members on a genome-wide level. RNA of *Salmonella* cultures treated for 3 h with 10 µg/mL of BPIFB was isolated and subjected to strand-specific sequencing. Bacteria exposed to the BPIFB buffer PBS served as control under identical conditions. To our surprise, only four genes exhibited a statistically significant, greater than 2-fold upregulation and two non-coding RNAs (ncRNA) showed significant downregulation in the BPIFB-treated samples (**Figure 3B** and **Supplementary Table 1**). The identified upregulated genes *proV*, *proW* and *proX* form the ProU (*proVWX*) operon encoding for a high-affinity glycine betaine transport system involved in osmoregulation (Stirling et al., 1989). STM14_1968 encodes for a putative S-(hydroxymethyl) glutathione dehydrogenase/class III alcohol dehydrogenase. The ncRNA STnc3740 (SLnc1015) is bound by ProQ and might act as a *cis*-antisense RNA (Smirnov et al., 2016) while the function of *rydB* (*tpe7*, IS082) is not known (Wassarman et al., 2001). We addressed the possible role of ProU for the observed phenomenon by generating a mutant. No impact of ProU on the ability of the cells to adhere after stimulation with 5 µg/mL BPI could be observed (**Figure 3C**).

With no significant transcriptional changes detectable upon exposure to BPIF proteins, we opted to elucidate the role of protein *de novo* synthesis for the observed phenotype. While treatment of the bacteria with chloramphenicol during stimulation with BPI did completely inhibit bacterial growth (data not shown), it had only a minor effect on the adhesion of *S*. Typhimurium (**Figure 3D**). From these experiments, we concluded that transcriptional reprogramming and protein *de novo* synthesis are not involved in BPIF protein-mediated bacterial adhesion.

### BPIF protein-mediated adhesion depends on pre-formed type 1 fimbriae

**Figure 4:**
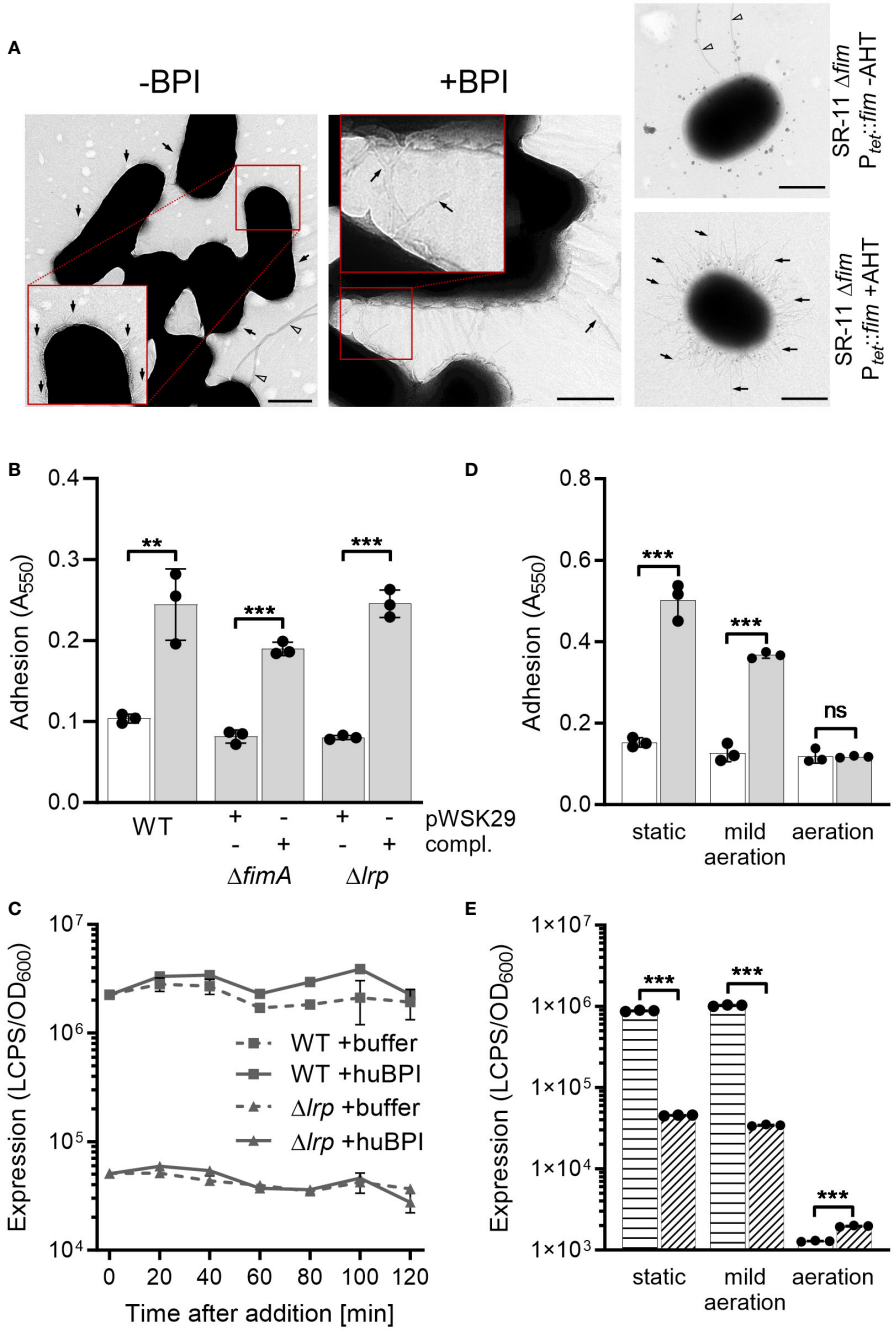
BPIF-induced adhesion is mediated by type 1 fimbriae. **(A)** Transmission electron microscopy (TEM) of *S*. Typhimurium wild type (STM WT) bacterial suspensions without **(-)** or with (+) addition of 10 µg/mL rhuBPI. A strain with inducible expression of type 1 fimbriae (SR-11 Δ*fim* P*_tet_*::*fim*) was included as negative or positive control (+AHT). Arrows indicate type 1 fimbriae where arrowheads designate flagella. Scale bar = 500 nm. **(B)** Adhesion of different strains to plastic surface after treatment for 2 h with 1 µg/mL huBPI (grey bars) or BPI buffer (white bar) was quantified by crystal violet staining. **(C)** *FimA* promoter activity in STM WT (squares) and Δ*lrp* (triangles) strains was quantified after addition of huBPI (solid line) or buffer (hatched line) using a luminescence reporter. Data normalized to bacterial density (OD_600_) is shown. **(D)** STM WT was grown O/N under the conditions as indicated. After treatment for 2 h with 1 µg/mL huBPI (grey bars) or BPI buffer (white bar) adhering bacteria were quantified by crystal violet staining. **(E)** The STM WT (horizontal hatched) and the Δ*lrp* (diagonal hatched) reporter strains from **(C)** were grown under the same conditions as in **(D)** and luminescence activity was quantified. The results shown are presented as mean ± SD of one representative experiment out of three independent experiments done in triplicates. Statistical significance was calculated using a two-tailed unpaired Student’s *t* test and was defined as ** for *p* < 0.01, *** for *p* < 0.001, ns, not significant.

Our previous results demonstrated that BPIF proteins bind to the bacterial surface and BPIF protein-induced adhesion is a post-translational process. Therefore, we reasoned that bacterial binding might rely on existing bacterial surface structures and performed transmission electron microscopy (TEM) in order to detect such structures. Under both conditions, untreated and huBPI-exposed, thin proteinaceous appendages were observed on the STM cell envelope (**Figure 4A**). Similar surface structures were observed using a strain with inducible expression of type 1 fimbriae (**Figure 4A**). Next, mutants deficient for *fimA* or *lrp* genes were generated. While *fimA* encodes for the major pilin subunit of type 1 fimbriae, Lrp activates the transcription of the *fim* operon (McFarland et al., 2008). Both mutants did not adhere in the presence of huBPI. However, complementation of the knockouts by plasmids harboring the deleted genes did restore the phenotype (**Figure 4B**). Identical results were obtained using knockouts of the tip adhesin FimH and the usher FimD (data not shown).

Next, we monitored the activity of the *fimA* promoter (P*_fimA_*) over time upon exposure to huBPI using a transcriptional fusion to a bacterial luciferase operon as reporter. Consistent with our transcriptome data, P*_fimA_* activity changed neither significantly over time nor upon addition of huBPI (**Figure 4C**). As previously described (McFarland et al., 2008), *fimA* promoter activity was strongly reduced in a Lrp-deficient strain (**Figure 4C**). Because transcriptional regulation and protein *de novo* synthesis are not required for the adhesion phenotype, we concluded that fimbriae have to be pre-formed to enable the effect. To address this assumption experimentally, bacteria were cultured under conditions known to induce and to repress expression of fimbriae (Saini et al., 2010). Under static growth or mild aeration, both conditions known to induce *fim* gene expression, huBPI was able to induce bacterial adhesion. However, no adhesion was observed when *Salmonella* were grown fully aerated which represses Fim transcription (**Figure 4D**). In line with that, repression of *fim* gene expression was observed for bacteria grown fully aerated using a luciferase reporter fusion. However, high luciferase activity was detected for statically grown or low aerated cultures (**Figure 4E**). As expected, deletion of *lrp* reduced *fimA* promoter activity under inducing culture conditions (**Figure 4E**).

### Modification of fimbrial adhesion specificity by BPIF proteins

**Figure 5:**
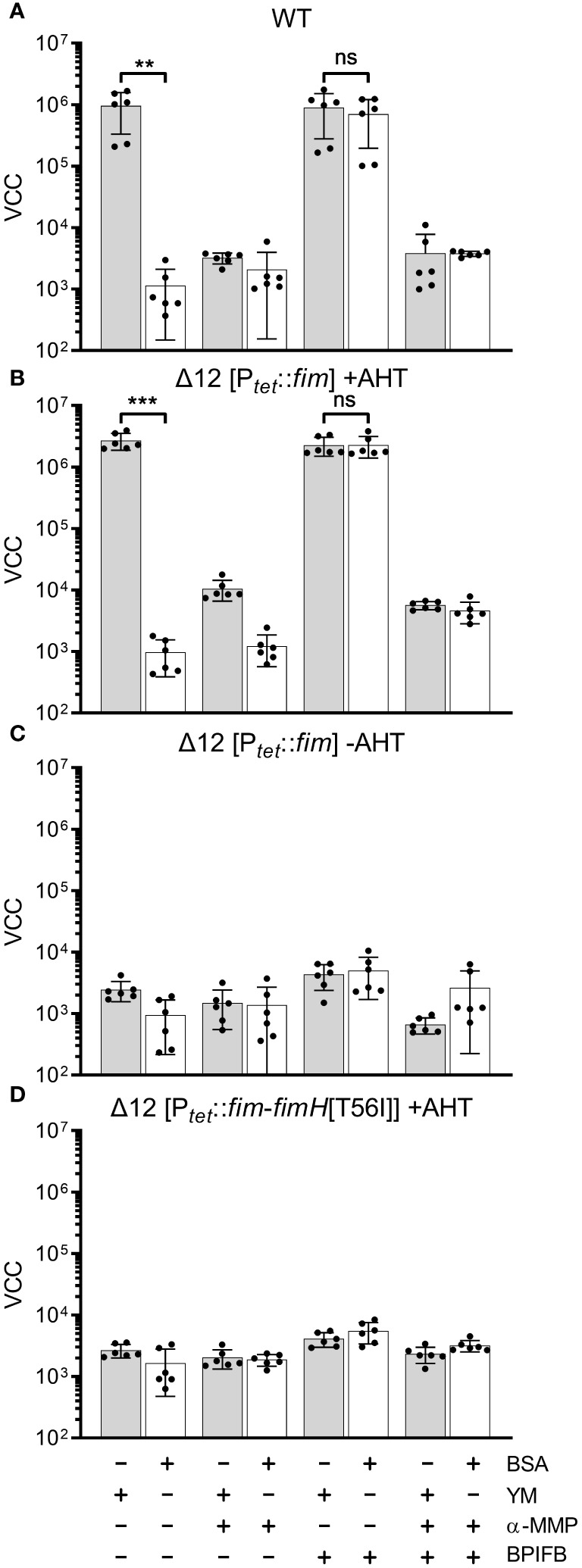
FimH-mediated mannose binding is required for BPIF-induced adhesion. **(A-D)** *S*. Typhimurium SR-11 wild type (WT) **(A)**, Δ12 [P*_tetA_*::*fim*] **(B, C)** and Δ12 [P*_tetA_*::*fim*-*fimH*[T56I]] **(D)** were allowed to adhere to yeast mannan (YM) coated (grey bars) or BSA treated (open bars) wells in the presence of 10 µg/mL BPIFB and/or 1% α-MMP (α-MMP) as indicated. Expression of WT fimbriae from P*_tetA_*::*fim* was either induced (+AHT) **(B)** or left uninduced **(C)**. In **(D)**, expression of fimbriae containing a T56I mutated FimH was induced. Bound bacteria were quantified using virtual colony counts (VCC) as described in material and methods. The results shown are mean ± SD of 6 independent experiment done in 4-fold replicates. Statistical significance was calculated using a two-tailed unpaired Student’s *t* test and was defined as ** for *p* < 0.01, *** for *p* < 0.001, ns, not significant.

In our experimental setting bacterial adhesion to plastic surfaces was depending on the presence of type 1 fimbriae in conjunction with a protein of the BPIF family. Therefore, we hypothesized that the BPIF proteins bind to the type 1 fimbriae that facilitates bacterial adhesion. Type 1 fimbriae exhibit a lectin-like activity through recognition of and subsequent binding to mannose moieties via the tip adhesin FimH (Humphries et al., 2003). This results in mannose sensitivity e.g. binding can be abrogated with excess of soluble ligand (Ofek et al., 1977). In order to test whether BPIF protein-induced adhesion of *Salmonella* is dependent on the interaction between type 1 fimbriae and mannose, wells were either coated with mannose-containing yeast mannan (YM) or bovine serum albumin (BSA) as a control. To increase the resolution and dynamic range of the bacterial adhesion assays, we quantified surface-bound bacteria based on virtual colony counts (VCC) (Hoffmann et al., 2018). As expected, strong bacterial binding was observed for STM SR-11 WT on YM-coated surfaces, but not on BSA-coated wells (**Figure 5A**). However, with addition of the BPIF family member BPIFB (LPLUNC1), bacteria started to bind to the BSA-coated wells to a similar extent than to YM-coated wells. Addition of the non-metabolizable α-D-mannose derivative methyl α-D-mannopyranoside (α-MMP) (Ofek et al., 1977) abrogated bacterial adhesion independent of the well surface coating and addition of BPIFB (**Figure 5A**). Similar results were obtained using a fimbriae-deficient background (Δ12) of *S*. Typhimurium SR-11 where expression of the *fim* operon was induced from a plasmid with addition of AHT (**Figure 5B**). No adhesion was observed when AHT was omitted and the *fim* operon was left uninduced (**Figure 5C**).

Because BPIF protein-induced adhesion was mannose sensitive, the mannose binding capacity of FimH seems to play an essential role. To test this, we introduced a point mutation (T56I) into FimH of the *fim* expression plasmid which renders FimH unable to interact with mannose residues (Kisiela et al., 2012). Because FimH is required to initiate the top-to-bottom synthesis of type 1 fimbriae, deletion of the gene inhibits Fim biogenesis (Munera et al., 2007). Therefore, we checked proper expression of type 1 fimbriae through flow cytometric detection of surface-localized FimA. Here, no differences in FimA expression were observed for plasmids harboring either WT FimH or T56I FimH (**Supplementary Figure 2**). Induction of expression of the FimH T56I mutant operon neither resulted in binding to YM nor to BSA in the presence of BPIFB (**Figure 5D**).

Although we could demonstrate the functional expression of fimbriae containing the FimH mutant T56I on the bacterial surface, only fimbriae with functional FimH were able to bind to YM-coated surfaces and sustain the effects of BPIF proteins. The importance of mannose binding was illustrated by the fact that the soluble ligand α-MMP completely inhibited not only binding to YM but also BPIF protein-induced adhesion of STM. If the surface of the plate was covered with a mannose-containing fimbrial ligand (YM), adhesion of the bacteria was BPIF protein independent. In the absence of such surface ligands (BSA), addition of BPIF proteins promoted adhesion by altering the binding specificity of the bacteria depending on functional fimbriae.

### Mannose-containing glycostructures are essential for interaction of BPIF proteins with fimbriae

**Figure 6:**
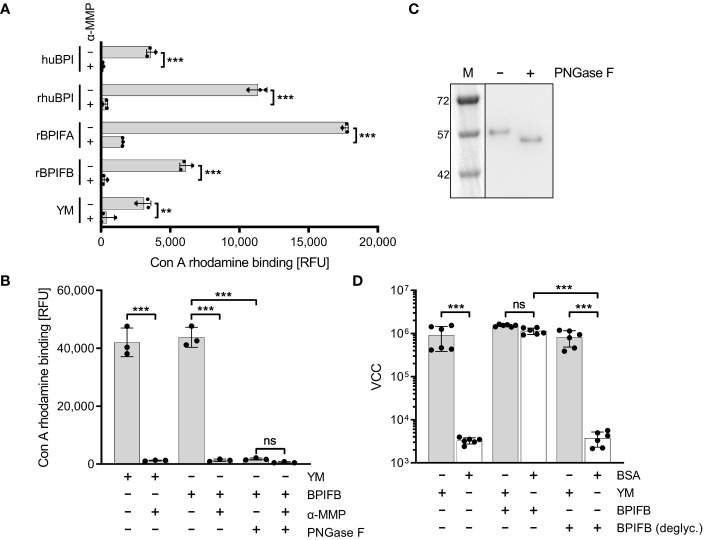
Mannosylation of BPIF proteins is required to mediate adhesion of *S*. Typhimurium. **(A)** Mannosylation of BPI isolated from human neutrophils (huBPI), recombinantly produced BPIF proteins or yeast mannan (YM) as indicated was tested through binding of the fluorescently labeled mannose-specific lectin concanavalin A (Con A rhodamine). Mannose sensitivity of Con A rhodamine binding was tested with addition of 1% α-MMP. **(B)** In a similar assay as shown in **(A)**, the effect of PNGase F-mediated deglycosylation of BPIFB is depicted. **(C)** Western blot analysis of PNGase F treated (+) or mock treated **(-)** BPIFB protein. **(D)** VCC-based quantification of bacteria adhering to YM-coated (grey bars) or BSA-treated (open bars) surfaces. The results shown are mean ± SD of 3 **(A, B)** or 6 **(D)** independent experiments done in 4-fold replicates. Statistical significance was calculated using a two-tailed unpaired Student’s *t* test **(A)** or one-way ANOVA **(B, D)** and was defined as ** for *p* < 0.01 and *** for *p* < 0.001, ns, not significant.

It is known that members of the BPIF family are glycosylated (Bingle et al., 2010; Ghafouri et al., 2004; Kohlgraf et al., 2012; Weersink et al., 1993). Therefore, we reasoned that these proteins might directly bind to FimH due to their glycosylation patterns. We showed that indeed all our recombinantly expressed proteins (rhuBPI, rBPIFA, rBPIFB) as well as BPI isolated from human neutrophils (huBPI) are bound by rhodamine-labeled concanavalin A (Con A), a lectin with mannose binding specificity (**Figure 6A**). Labeling of the proteins was mannose sensitive as demonstrated with addition of 1% α-MMP that abrogated lectin binding completely (**Figure 6A**). In order to analyze the dependence of rBPIFB or rhuBPI sugar moieties for the interaction with FimH, we de-glycosylated the proteins via treatment with N-glycosidase F (PNGase F) which efficiently removes N-linked glycans from asparagine residues. With PNGase F treatment Con A rhodamine binding to BPIFB was reduced almost to background level (**Figure 6B**). Furthermore, PNGase F-treated BPIFB migrated faster in gel electrophoresis compared to a mock-treated control (**Figure 6C**). Similar results but to a somewhat lower extent were obtained using huBPI (**Supplementary Figures 3A, B**). Further functional testing of deglycosylated BPIFB revealed that the protein completely lost its adhesion-inducing potential towards STM (**Figure 6D**).

To identify the part of the BPI protein mediating FimH binding we produced an N-terminally truncated version of BPI as a recombinant protein. This C-terminal part of BPI which neither shows LPS neutralizing activity nor antimicrobial effects [(Weiss et al., 1978) and data not shown] was still able to induce *Salmonella* adhesion, albeit to a lesser extent than full length BPI (**Supplementary Figure 4**). Therefore, the adhesion-inducing potential of BPI/BPIFB depends on mannose-containing glycostructures and is probably independent of the LPS binding capacity of the protein.

### BPI can promote *Salmonella* adherence to cultured cells

**Figure 7:**
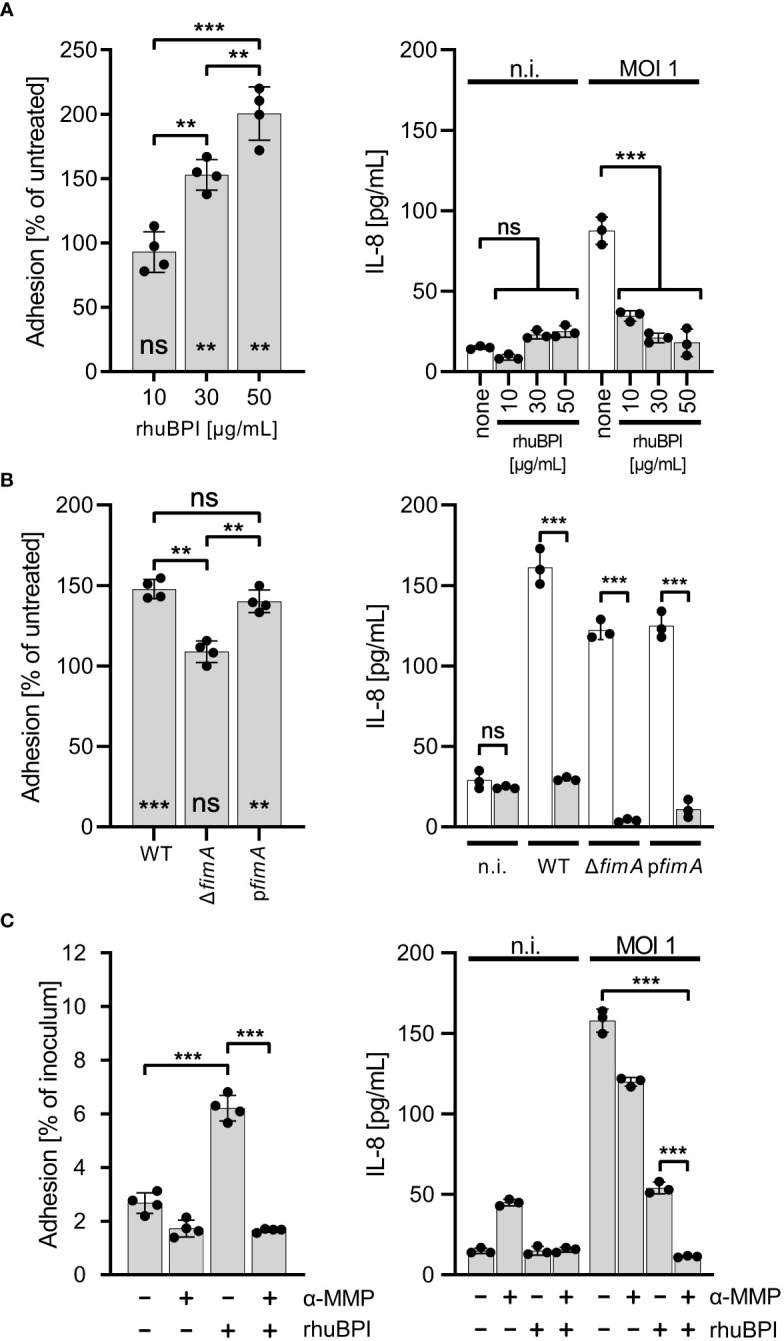
BPI promotes cellular adhesion of *Salmonella* to BEAS-2B cells. **(A)** Cells were infected for 1.5 h at a multiplicity of infection of one (MOI 1) with *S.* Typhimurium (STM) WT which was pre-incubated for 30 min. with 10, 30 or 50 µg/mL recombinant human BPI (rhuBPI). The left panel shows the % adhered bacteria normalized to untreated controls. In the right panel, cells were infected as described above, but subsequently treated O/N with gentamicin and IL-8 was quantified from supernatants. As controls, uninfected (n.i.) cells were treated with similar amounts of rhuBPI as indicated or left untreated (none). **(B)** The experiments as explained in **(A)** were performed with STM WT, Δ*fimA* and p*fimA* (plasmid-complemented) with pre-incubation of the bacteria using 50 µg/mL rhuBPI. **(C)** The experiments as explained in **(A)** were done with or without pre-incubation of STM WT with 50 µg/mL rhuBPI and/or methyl α-D-mannopyranoside (α-MMP). The results shown are mean ± SD of one representative experiment out of three performed done in quadruplicates. Statistical significance was calculated using one-way ANOVA for the comparisons as indicated and using one sample t-test against the theoretical mean of 100 for left panels of **(A, B)**. Significance was defined as ** for *p* < 0.01, *** for *p* < 0.001 and ns, not significant.

It has been shown that type 1 fimbriae can mediate adhesion to mannosylated surface structures of M-cells (Hase et al., 2009) and some epithelial cell lines (Bäumler et al., 1996).Therefore, we were interested whether the altered adhesion specificity through fimbriae-bound BPIF proteins not only enables binding to abiotic surfaces but would also promote bacterial adhesion in a cellular assay. As a model, we used the human bronchial epithelial cell line BEAS-2B which can be readily infected by *Salmonella*, and releases IL-8 upon infection with *Salmonella* (data not shown and **Figure 7**). BEAS-2B cells were infected with STM pre-incubated with or without rhuBPI for 1.5 h. The functionality of rhuBPI in this experimental setting could be demonstrated by the reduced amount of IL-8 released from the infected cells due to its LPS-neutralizing activity (Wittmann et al., 2008) (**Figure 7**). Pre-incubation with rhuBPI led to an increase of adherence of STM WT bacteria in a dose-dependent manner (**Figure 7A**). In contrast, STM Δ*fimA* pre-incubated with 50 µg/mL rhuBPI was unresponsive to the treatment indicating that the effect is also depending on type 1 fimbriae function in this setting. Complementation of this mutant by a plasmid harboring the *fimA* gene let the strain regain its rhuBPI-dependent adhesion (**Figure 7B**). To further interfere with fimbriae-dependent adhesion, α-MMP was added during the experiment. The incubation with α-MMP drastically reduced the rhuBPI-mediated enhanced adhesion of *Salmonella* in BEAS-2B cells (**Figure 7C**) suggesting a mechanism in this model similar to that observed for binding to abiotic surfaces.

### Fimbrial binding reduces bactericidal effects of BPI

**Figure 8:**
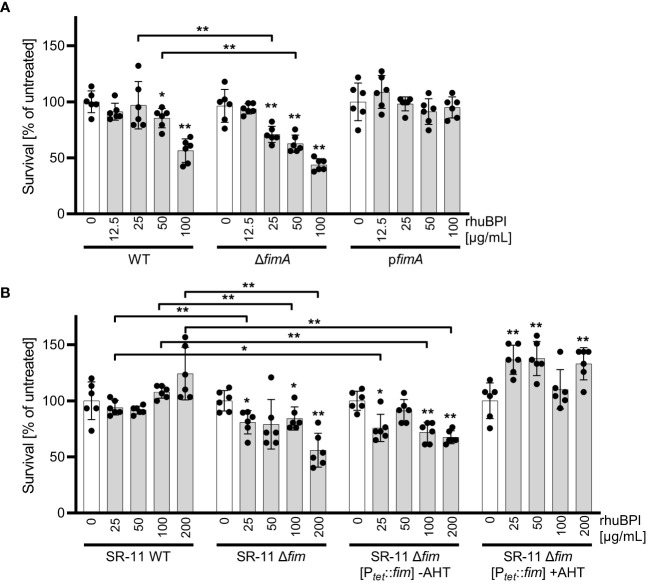
The presence of type 1 fimbriae reduces antimicrobial activity of BPI. **(A)** Bacterial survival after one hour of incubation with the indicated concentrations of rhuBPI (grey bars) or buffer controls (open bars) was quantified for strains *S*. Typhimurium (STM) WT, Δ*fimA* and p*fimA* (plasmid-complemented) by plating. **(B)** Bacterial survival of *S*. Typhimurium strain SR-11 WT, Δ*fim* and Δ*fim* [P*_tet_*::*fim*] (inducible *fimAICDHF* expression) was quantified as described in **(A)**. The results are presented as mean ± SD of data normalized to buffer controls of five independent experiments. Samples were compared against the respective buffer controls or between test strains as indicated. Statistical significance was calculated using Mann Whitney test and was defined as * for *p* < 0.05, ** for *p* < 0.01.

We could show that BPIF family proteins are bound by *Salmonella* through type 1 fimbriae and this binding enhances adhesion capabilities of the bacteria. To exert its antimicrobial activity, BPI has to interact with the bacterial OM, presumably through LPS binding. Given the moderate antimicrobial activity of BPI against STM (**Figure 1A**) we speculated that fimbriae could sequester BPI to prevent its binding to the bacterial OM. Therefore, we exposed STM WT and bacteria deficient for *fimA* to increasing concentrations of rhuBPI. While both strains showed a dose-dependent killing, the effect was significantly stronger for the *fimA* mutant (**Figure 8A**). Interestingly, plasmid-based complementation using the native promoter (p*fimA*) completely abrogated the antimicrobial effect of rhuBPI (**Figure 8A**). When we tested the *Salmonella* WT strain SR-11 in a similar assay, we observed no antimicrobial effect up to 200 µg/mL rhuBPI. In contrast, an isogenic strain lacking the *fim* operon showed significantly reduced bacterial survival. Similar results were obtained when a plasmid for inducible expression of the *fim* operon was left uninduced in strain SR-11 Δ*fim*. However, when expression of type 1 fimbriae was induced, survival under all concentrations of rhuBPI exceeded that of the buffer control (**Figure 8B**). In summary, we could demonstrate a protective effect of fimbriae against the antimicrobial action of BPI. Although strain-specific differences exist, isogenic mutants deficient for type 1 fimbriae were more susceptible compared to the respective WT strains.

## Discussion

**Figure 9:**
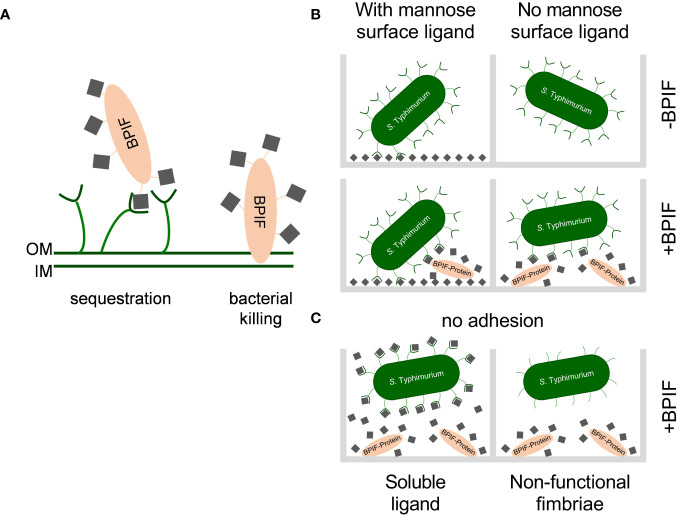
Working models for consequences of the interaction between BPIF-proteins and type 1 fimbriae. **(A)** Model for the protective effect of type 1 fimbriae against the bactericidal activity of BPIF proteins. Left: In the presence of type 1 fimbriae the BPIF protein is sequestered through Fim-mediated binding of mannose residues (grey squares). Right: In the absence of fimbriae BPIF proteins interact with LPS on the OM which subsequently leads to membrane rupture. IM, inner membrane; OM, outer membrane. **(B)** *Salmonella* readily binds mannosylated (grey squares) surfaces via type 1 fimbriae (left panels). However, in the absence of surface-exposed mannose, glycosylated BPIF proteins can mediate bacterial binding as fimbrial ligands thereby expanding the binding specificity of *Salmonella* (lower right panel). **(C)** The interaction between fimbriae and BPIF proteins is mannose sensitive (left panel) and requires functional fimbriae (right panel).

*Salmonella* is a versatile pathogen which has evolved multiple pathways to recognize and respond to the presence of AMPs. Antimicrobial peptides are host derived factors with cationic properties and are thought to interact with the mainly negatively charged bacterial cell wall. A common countermeasure to reduce the affinity of the bacterial cell envelope for AMPs is to lower the negative net charge through modifying LPS with positively charged moieties (Peschel and Sahl, 2006). For *Salmonella*, the PmrAB TCS was shown to induce such LPS modifications in the presence of BPI and other AMPs which in turn prevented binding of larger amounts of these peptides (Farley et al., 1988; Gunn et al., 2000). Moreover, Farlay et al. demonstrated that *Salmonella* expressing a smooth form of LPS were more resistant to the antimicrobial activity of BPI most likely due to a decreased binding affinity (Farley et al., 1988). This is in line with older data which showed differential susceptibility of *Salmonella* strains against BPI depending on the expression of LPS variants (Weiss et al., 1978). Our data suggest another resistance mechanism depending on type 1 fimbriae where mannosylated BPI is bound by the fimbrial tip adhesin thereby preventing further interaction with LPS of the outer membrane (**Figure 9A**). Here, the somewhat lower *K_D_* of 1-2 × 10^-6^ M of the (high affinity) FimH-mannose interaction (Aprikian et al., 2007) compared to a *K_D_* of 2-5 × 10^-6^ M for the BPI-LPS interaction (Gazzano-Santoro et al., 1992) could lead to AMP sequestration by fimbriae (**Figure 9A**). Additionally, AMP-decorated fimbriae could further sterically block access to LPS for larger molecules.

High concentrations of BPI are required to exert an antimicrobial effect on *Salmonella* as reported by this and other studies (Weiss et al., 1978; Farley et al., 1988; Roland et al., 1993). Because these concentrations might not always be reached *in vivo*, we also applied BPI well below its inhibitory concentration. Using this setup, we still observed significant binding of BPI to the bacterial surface. Interestingly, there was less intense LPS staining (**Figure 1B**) for BPI-treated bacteria which could be due to steric hindrance or LPS modification (see above), both resulting in reduced anti-LPS antibody binding. Importantly, addition of sub-inhibitory concentrations of BPIF family members promoted *Salmonella* adhesion. Mechanistically, pre-formed type 1 fimbriae were sufficient to trigger adhesion with transcriptional reprogramming excluded by RNA-seq analysis. Hence, we propose that sub-antimicrobial concentrations of BPI do not trigger the AMP sensing systems which was supported by the dispensability of one or a combination of the PhoPQ-, PmrAB-, Rcs-, or CpxRA systems.

Type 1 fimbriae of *Salmonella* can bind mannose-containing, glycosylated moieties of BPIF family members BPI, LBP, BPIFA and BPIFB via their tip adhesin FimH. This resulted in altered binding specificity of the bacteria to additionally support adhesion to previously low-binding abiotic or cellular surfaces. While surfaces with exposed mannose residues were bound in a type 1 fimbriae-dependent but BPIF protein-independent manner, Fim-bound BPIF proteins can mediate binding to non- or low-mannosylated surfaces (**Figure 9B**). Depending on the binding affinities, it is also conceivable that the BPIF proteins bind the respective surface first with type 1 fimbriae-dependent bacterial binding to follow. However, independent of the order of binding the result will be similar. As expected, BPIF protein-dependent adhesion is mannose sensitive and requires functional type 1 fimbriae (**Figure 9C**). Besides increased adhesion to abiotic surfaces, we also observed elevated numbers of adhered bacteria pretreated with BPI to BEAS-2B cells (**Figure 7**). For *Shigella flexneri*, binding of the α-defensin HD5 to membrane proteins enabled the bacteria to adhere to epithelial cells and to mediate pathogenicity. In contrast to our findings, the binding of HD5 to the bacterial membrane was independent of fimbriae expression since Con A failed to inhibit HD5-enhanced *in vitro* infection (Xu et al., 2018).

Our results with BEAS-2B cells suggested that there might be a limited availability of terminal mannose residues on the surface of these cells. Here, the bridging effect of rhuBPI was able to compensate for that in WT and plasmid-complemented Δ*fimA* bacteria (**Figure 7B**). Type 1 fimbriae are important players in the pathogenicity of *Salmonella* with their contribution to the bacteria host interaction likely depending on the amount of surface mannose available. For example, type 1 fimbriae foster the reversible binding of *Salmonella* to HeLa cells during the invasion process (Misselwitz et al., 2011) and the uptake of STM or *E. coli* by dendritic cells (Guo et al., 2007). Fim-mediated adhesion was also shown to promote biofilm formation (Boddicker et al., 2002). It is tempting to speculate that the increased binding affinity, especially for abiotic, non-mannosylated surfaces, of BPIF protein-decorated fimbriae could result in subsequent biofilm formation.

While demonstrating a positive effect of BPIF proteins on adhesion, surface binding of host-derived proteins could also have detrimental effects on the bacteria. For example, binding of uromodulin to uropathogenic *E. coli* inhibits bacterial adhesion to bladder epithelial cells (Conover et al., 2016). Previously, surface binding of BPI was shown to promote phagocytotic uptake of bacteria by neutrophils and monocytes which was attributed to the binding of BPI to LPS (Iovine et al., 1997). Besides LPS, additional ligands of BPI have been identified. It was demonstrated that BPI is able to interact with the surface of Influenza A virus. Although the exact ligand could not be determined it is clear that it will not be LPS (Pinkenburg et al., 2016). Furthermore, BPI was able to enhance the stimulating capacity of bacterial lipoproteins (Bülow et al., 2018). Here we add an additional ligand of BPI to the list since we show that BPI interacts with FimH.

Expression of type 1 fimbriae by bacterial pathogens can be a double-edged sword. Despite mediating adhesion to various host cells and thereby enabling further steps in the pathogenesis, fimbriae are structures recognized by the host immune system. Specifically, the fimbrial tip protein FimH has been implicated in sensing of bacterial pathogens by M-cells via direct binding to the GP2 protein (Hase et al., 2009). Lack of FimH reduced antigen-specific immune responses in Peyer’s patches because of decreased transcytosis through M-cells (Hase et al., 2009). As a consequence, bacteria need to regulate expression of fimbrial operons tightly *in vivo* to balance between adhesion function and exposure of a target structure for the host adaptive immune system (Humphries et al., 2003; Klaffenbach et al., 2011). Our results suggest the presence of an additional layer to fine-tune the activity of type 1 fimbriae via interaction with host cell-derived glycosylated proteins. Here binding of certain proteins belonging to the innate immune system of the host to the bacterial fimbriae serves as a bridge to enable Fim-mediated adhesion to a broader range of (cellular) surfaces. In case of BPI we showed that fimbriae can act as scavengers thereby reducing the antimicrobial activity of the protein. Further experiments are needed to answer the question whether these results can be transferred to other *Salmonella* fimbrial operons or other fimbriated bacteria.

## Materials and methods

### Bacterial strains and plasmids

Bacterial strains used in this study are listed in **Table 1**. *Salmonella enterica* serovar Typhimurium (STM) NCTC 12023 was used as a wild type (WT) strain. Isogenic mutants from strain NCTC 12023 were generated by λ Red-mediated recombination as described before (Blank et al., 2011; Chakravortty et al., 2002) using template plasmids pKD4 (Datsenko and Wanner, 2000) or pWRG100 (Blank et al., 2011) with primers as detailed in **Supplementary Table 2**. The kanamycin resistance cassette was optionally removed by expression of Flp recombinase from plasmid pCP20 (Cherepanov and Wackernagel, 1995) or a second λ Red recombination step with I-SceI selection (Blank et al., 2011). For complementation of *fimA* and *lrp* mutants, fragments containing the native promoter and the cognate coding sequence were PCR-amplified from *S*. Typhimurium genomic DNA using forward primers containing an EcoRI site and reverse primers with an XbaI-site. Fragments were cloned in pWSK29 using EcoRI and XbaI to generate pWRG79 (p*fimA*) and pWRG80 (p*lrp*). The transcriptional *fimA* luminescence reporter plasmid pWRG114 was constructed based on pGEN-luxCDABE (Lane et al., 2007). For that, the *fimA* promoter was amplified by PCR using primers Pro-FimA-EcoRI-for and Pro-FimA-SmaI-rev with genomic DNA as template and subsequently cloned in pGEN-luxCDABE via EcoRI/SmaI. All constructs were initially screened by colony PCR using suitable check primers and finally verified by restriction analysis and sequencing.

**Table 1:** Bacterial strains and plasmids used in this study.

Strain or plasmid	Relevant characteristic(s)	Source or Reference
*Pseudomonas aeruginosa* ATCC 27853	wild type; reference strain	ATCC
*S. enterica* serovar Typhimurium strains		
NCTC 12023	wild type	NCTC, Colindale, UK
SR-11	wild type	(Schneider and Zinder, 1956)
SR-11 Δ*fim*	Δ*fimAICDHF*	(Elpers et al., 2020)
SR-11 Δ12	Δ*fim* Δ*stb* Δ*sth* Δ*stf* Δ*sti* Δ*bcf* Δ*saf* Δ*pef* Δ*stc* Δ*stj* Δ*lpf*::*aph*	(Elpers et al., 2020)
MvP881	*csgD*::*aph*, Kan^r^	This study
MvP897	*csgD* FRT	This study
WRG6	Δ*phoQ*	(Blank et al., 2011)
WRG9	*lrp*::*aph*, Kan^r^	This study
WRG10	*fimA*::*aph*, Kan^r^	This study
WRG13	*lrp* FRT	This study
WRG14	*fimA* FRT	This study
WRG23	*phoQ*-T48I, PhoP^c^ phenotype	(Blank et al., 2011)
WRG46	*rcsD*::*aph*, Kan^r^	This study
WRG110	*cpxRA*::*aph*, Kan^r^	This study
WRG111	*pmrAB*::*aph*, Kan^r^	This study
WRG116	*cpxRA* FRT	This study
WRG117	*pmrAB* FRT	This study
WRG123	Δ*phoQ pmrAB*::*aph*, Kan^r^	This study
WRG345	*proVWX*::I-SceI::*aph*, Kan^r^	This study
WRG349	Δ*proVWX* (ΔProU)	This study
Plasmids		
P*_tet_*::*fim* (p4392)	*tetR* P*_tetA_*::*fimAICDHF*	(Hansmeier et al., 2017)
pKD46	Red-expressing plasmid, temperature sensitive, Amp^r^	(Datsenko and Wanner, 2000)
pKD4	Red template plasmid, suicide vector (ori R6K), Amp^r^, Kan^r^	(Datsenko and Wanner, 2000)
pCP20	Flp recombinase expression plasmid, temperature sensitive, Amp^r^	(Cherepanov and Wackernagel, 1995)
pGEN-luxCDABE	P_EM7_::*luxCDABE*, Amp^r^	(Lane et al., 2007)
pWSK29	Low-copy number vector, Amp^r^	(Wang and Kushner, 1991)
pfimA (pWRG79)	P*_fimA_*::*fimA* in pWSK29, Amp^r^	This study
plrp (pWRG80)	P*_lrp_*::*lrp* in pWSK29, Amp^r^	This study
pWRG99	pKD46 (Datsenko and Wanner, 2000) with tetracycline-inducible I-SceI, Amp^r^	(Blank et al., 2011)
pWRG100	pKD3 (Datsenko and Wanner, 2000) with I-SceI recognition site, Cm^r^	(Blank et al., 2011)
pWRG114	P*_fimA_*::*luxCDABE* in pGEN-luxCDABE, Amp^r^	This study
P*_tet_*::*fim-fimH*[T56I] (pWRG934)	*tetR* P*_tetA_*::*fimAICDH*[T56I]*F*	This study
pMT/BiP/V5-His A hygromycin B	Recombinant expression in D. mel-2 cells	This study

### Expression of recombinant BPI, BPIFA, BPIFB, LBP, procryptdin

The coding sequence without the respective leader signal of these proteins and peptides was amplified by PCR with the primers listed in **Table 1**. With the appropriate restriction enzymes the purified PCR fragments as well as the expression vector pMT/BiP/V5-His (Thermo Fisher Scientific) which we have altered that it harbored a hygromycin B resistance cassette in order to select in insect cells were digested and ligated thereafter. The expression plasmid will lead to a recombinant protein with a *Drosophila* leader for secretion and a V5-His tag for purification as well as detection purposes. The sequence verified vector was transfected into *Drosophila melanogaster* 2 (D. mel-2; Thermo Fisher Scientific) cells by Cellfectin (Thermo Fisher Scientific) and the transfected cells were propagated in SPODOPAN (PAN Biotech, Aidenbach, Germany). 72 h after the transfection cells carrying the plasmid were selected by the addition of hygromycin B. The expression of the recombinant protein was induced by the addition of 500 µM CuSO_4_ for 4 days and the supernatant was collected. The recombinant protein was purified from the supernatant on a HiTrapTM Chelating HP column (GE Deutschland) and eluted by an imidazole gradient using an Äkta FPLC (GE Deutschland). After the imidazole had been removed by dialysis against PBS pH 7.4 the purity and the specificity of the protein were analyzed by SDS-PAGE followed by Coomassie staining as well as Immunoblot. The purity of the proteins was always > 90%.

### Antimicrobial activity of BPI

Antimicrobial activity of BPI was determined in 3 to 5 independent experiments essentially as described before (Aichele et al., 2006). Briefly, *S*. Typhimurium and *Pseudomonas aeruginosa* were grown over night (O/N) in LB or TSB, respectively. After re-inoculating the strains 1:100 in fresh medium, cultures were allowed to grow to mid log phase (~2.5 h) and washed twice in PBS (PAA, Pasching, Austria). Assays were performed in 96-well microtiter plates (#655180, Greiner Bio-One, Frickenhausen, Germany) using 10,000 CFU/mL and the indicated amount of BPIF protein or buffer control in a total volume of 50 µL PBS per well. The plates were incubated for the indicated time points swimming in a water bath at 37°C. Thereafter, an aliquot of the bacteria was plated on LB-plates and enumerated the following day.

### Adhesion assay

Bacteria were inoculated in 4 mL LB and grown O/N either static or with mild agitation at 37°C. For crystal violet assays cultures were adjusted to an OD_600_ of 1.2 in LB and 100 µL of this inoculum was added to a cavity of a 96-well microtiter plate (Greiner Bio-One). For assays based on virtual colony counts (VCC), O/N cultures were washed twice in 1 mL PBS and finally adjusted to an OD_600_ of 0.5 in PBS. To inhibit protein synthesis, bacteria were incubated for 30 minutes in LB containing 50 µg/mL chloramphenicol prior addition of the AMP. Double-concentrated AMP or appropriate buffer was applied as described under biofilm assay except LB with 5 g/L NaCl was used. The plate was incubated in a humid chamber or in a water bath for various time points at 37°C. Where indicated, chloramphenicol at a concentration of 50 µg/mL was present during incubation. The absorbance at 600 nm (A_600_) was determined at the beginning and the end of an experiment using a M1000 plate reader (Tecan). Typically, 3 to 4 independent experiments were carried out.

### Biofilm assay

Two to three colonies of the freshly streaked strains were resuspended in PBS to an OD_600 =_ 0.15. The suspension was further diluted 1:100 in LB without salt and 100 µL of this inoculum was added to a cavity of a 96 well tissue culture-treated microtiter plate (Greiner Bio-One). To this suspension 100 µL of LB without NaCl containing double-concentrated recombinantly expressed AMP (see below), BPI isolated from human neutrophils (Athens Research & Technology, Athens, GA, USA) or the appropriate amount of control buffer (PBS or 50 mM Tris-HCl pH 7.0, 80 mM citrate/phosphate, 800 mM NaCl) was added. The plate was incubated in a humid chamber or swimming in a water bath for various time points at 28°C. The absorbance at 600 nm (A_600_) was determined at the beginning and at the end of an experiment using an Infinite M1000 plate reader (Tecan, Groedig, Austria). The biofilm data shown represents three independent experiments.

### Crystal violet staining

After removal of bacterial suspensions, wells were rinsed three times with 300 µL/well PBS. CV staining was carried out as described before (Kai-Larsen et al., 2010). Briefly, the plate was allowed to dry for at least 90 minutes and bound bacteria were stained by adding 200 µL/well filtered 3% (w/v) CV solution in H_2_O for 5 minutes at room temperature under mild agitation. Unbound CV was removed by rinsing 3 times with 200 µL/well H_2_O. The CV associated with bacteria was solubilized using 200 µL/well 96% ethanol with slow shaking for 20 minutes. The bound CV was quantified by measuring absorbance at 550 or 590 nm (A_550_ or A_590_) using an Infinite M1000 plate reader (Tecan).

### Virtual colony counts

Wells of a 96-well plate (Nunc Edge 2.0, ThermoFisher Scientific) were coated O/N either with 100 µL of 0.5 µg/mL yeast mannan (YM) (M3640, Sigma-Aldrich, Germany) or 0.1% bovine albumin fraction V (BSA) (Carl Roth, Germany) in PBS at room temperature. After washing 2× with 150 µL PBS, the wells were blocked for 30 min. with 170 µL 0.1% BSA in PBS at room temperature. After that, the wells were aspirated and 50 µL of PBS containing 0.2% BSA, double-concentrated BPIF proteins and optionally 2% of methyl α-D-mannopyranoside (α-MMP) (#67770, Sigma-Aldrich) was added. Fifty µL of the bacterial culture adjusted to OD_600 =_ 0.5 in PBS was added and the plates were incubated in a humid chamber for 4 h at 37°C. All wells were rinsed six times with 150 µL PBS to remove unbound bacteria. Surface-bound bacteria were quantified with virtual colony counts (VCC) based on growth curves recorded after addition of 150 µL brain heart infusion (BHI) medium supplemented with 1% α-MMP using a Multiskan Go or Sky plate reader (Thermo Fisher Scientific) with shaking and set to 37°C as described before (Hoffmann et al., 2018). All VCC data originates from six independent experiments done in quadruplicates.

### RNA-seq experiments

Bacterial O/N cultures were adjusted to an OD_600_ of 1.2 in LB and 10 µg/mL of recombinant murine BPIFB (rmLPLUNC1-His) in PBS or a similar amount of PBS only (buffer control) was added. After 3 h of incubation at 37°C in a water bath bacterial RNA was stabilized using the RNAprotect Bacteria Reagent (Qiagen, Hilden, Germany) and subsequently purified using the Total RNA Isolation Mini Kit (Agilent Technologies, Waldbronn, Germany). Absence of DNA was checked by PCR and suitable genome-specific primers. Equal amounts of RNA from three independent biological replicates were pooled and subjected to strand-specific RNA-seq (GATC Biotech, Koblenz, Germany). Raw data have been deposited with links to BioProject accession number PRJNA348183 in the NCBI BioProject database (https://www.ncbi.nlm.nih.gov/bioproject/) and have been uploaded to the Sequence Read Archive (SRA, https://www.ncbi.nlm.nih.gov/sra), accessions numbers SRR4420363 and SRR4420365. Reads were trimmed based on quality using Trimmomatic (Bolger et al., 2014). Rockhopper 2 (Tjaden, 2015) was used for mapping the reads to the concatenated sequence of strain *S*. Typhimurium ATCC 14028S and plasmid pSLT (accessions: CP001362 + CP001363). Differential expression analysis was done within Rockhopper 2 on the basis of extended annotation data including a set of non-coding RNAs described before (Kröger et al., 2013). Relative expression of the BPIFB- and PBS-treated samples was plotted using ggplot2 (Wickham, 2016) within R v3.5.1 (R Core Team, 2019).

### Luminescence assay

Either 200 µL/well as described under the adhesion assay or 100 µL/well O/N culture were directly quantified for luminescence activity in white 96 well microtiter-plates (Nunc, Langenselbold, Germany) using the Infinite M1000 plate reader (Tecan) in photon counting mode. Signals were integrated for 1 s and weakened with an OD1 filter if exceeding the detection limit. Absorbance at 600 nm was measured in parallel to account for differences in microbial growth. All experiments were done three times in triplicates.

### Test for mannosylation

Microtiter-plates (Nunc Maxisorp, ThermoFisher Scientific) were coated O/N at 4°C in a humid chamber with 50 µL of 5 µg/mL BPIF protein or YM in PBS. The wells were blocked for 1 h at room temperature with 200 µL/well 0.1% BSA in PBS. The wells were aspirated and 50 µL of 20 µg/mL rhodamine-ConA (Vector Labs, Peterborough, UK), optionally supplemented with 1% α-MMP, was added. After 1 h incubation in the dark at room temperature, the wells were washed 3× with 100 µL PBS + 0.05% Tween 20 (Sigma-Aldrich) and finally 50 µL PBS was applied to each well. Bound rhodamine-ConA was quantified using an Infinite M1000 plate reader (Tecan) in bottom read fluorescence mode set to 555 nm excitation- and 580 nm emission wavelengths. All experiments were done three times in triplicates.

### Fluorescence staining and confocal microscopy

Bacteria were bound to poly-L-lysine coated coverslips. *Salmonella* were stained using an anti-*Salmonella* group B antibody (BD Diagnostics, Heidelberg, Germany). BPI was detected using a monoclonal antibody (rat anti-Human BPI, obtained from HyCult, Leiden, The Netherlands). Primary antibodies were detected with AlexaFluor-coupled secondary antibodies (Invitrogen, Darmstadt, Germany). Images were acquired using a TCS NT (Leica Microsystems, Wetzlar, Germany) confocal laser-scanning microscope equipped with appropriate filter sets. Datasets were visualized using Imaris (Bitplane, Zurich, Switzerland) software package.

### Flow cytometry

Bacterial surface presentation of type 1 fimbriae was analyzed by flow cytometry using an Attune NxT (ThermoFisher Scientific) instrument. Bacterial cultures were treated as described before (Hansmeier et al., 2017). Briefly, bacteria (ca. 6 × 10^8^ cells) were harvested, washed once in PBS, fixed in 3% paraformaldehyde for 20 min and blocked with 2% goat serum in PBS for 30 min. Specific antiserum against FimA (goat-α FimA) was used diluted 1:1,000 in 2% goat serum in PBS and labeled with secondary goat-α-rabbit IgG antibody coupled to Alexa-Fluor488 diluted 1:2,000 in 2% goat serum in PBS. A mutant strain lacking type 1 fimbriae was always included as negative control for gating. Data were analyzed and visualized using Attune NxT software version 2.5 and the package “flowCore” within R v3.5.1.

### Transmission electron microscopy

Bacteria were treated as described above with 10 µg/mL rhuBP or buffer (PBS) but in the presence of 2% α-MMP. After 4 h of incubation at 37°C, bacteria were fixed with 2.5% glutaraldehyde in 0.1 M phosphate buffer, pH 7.2 for 30 min at RT. Afterwards, bacteria were concentrated by centrifugation at 1,000 × g for 5 min. and further fixed for 1 h by 2.5% glutaraldehyde in 0.1 M Na-cacodylate buffer, pH 7.2. Pellets were washed twice with H_2_O_dd_. Formvar/carbon-coated EM grids (100 mesh) were glow discharged immediately before use by an easiGlow instrument (Pelco) for 10 s with 15 mA. Of bacterial suspensions, 5 µL were dropped on EM grids and bacteria were negative-stained with 1% phosphotungstic acid at pH adjusted to 7.4. Visualization of type 1 fimbriae on bacterial surfaces was done by transmission electron microscopy (TEM) using a Zeiss Leo 902 system, operated at 80 keV. For more detailed information see (Hansmeier et al., 2017).

### Adhesion of *Salmonella* to BEAS-2B cells

The adhesion of *Salmonella* Typhimurium was analyzed in the BEAS-2B cell line (ATCC, CRL-9609). Therefore, 2 × 10^4^ BEAS-2B cells were plated with RPMI 1640 (PAN Biotech) complete (10% FCS, 50 U/mL penicillin, 50 µg/mL streptomycin, 2 mM glutamine, 0.25 mM 2-mercaptoethanol) in a 96-well plate and incubated O/N at 37°C and 5% CO_2_. Prior to infection with *Salmonella* the media was removed and replaced by RPMI 1640 medium 10% FCS without antibiotics. In the meantime, stationary *Salmonella* in suspension were diluted corresponding to a multiplicity of infection (MOI) of one and pre-incubated for 30 minutes with or without 10, 30, 50 µg/mL recombinant human BPI in RPMI 1640 medium 10% FCS without antibiotics. After that the BEAS-2B cells were infected with *Salmonella* for 1.5 h at 37°C and 5% CO_2_, cells were washed in PBS pH 7.4 and subsequently lysed in lysis buffer (PBS/0.3% Triton X-100). Bacteria were enumerated by plating serial dilutions of lysates on LB agar plates and subsequent counting of colony-forming units (CFU). The amount of the percentage of adhered bacteria was calculated in relation to the number of bacteria in the supernatant during this time point. To control for equal bacterial numbers in the experimental settings samples were collected from the inoculum, after the initial incubation of bacteria with or without BPI, and after incubation of bacteria for 1.5 h with the cells and plated on LB-agar plates. To investigate the contribution of type 1 fimbriae, WT, *fimA* mutant and complemented *Salmonella* strains were pre-incubated with 50 µg/mL recombinant BPI. Adhesion assay was carried out as described above either in the presence or absence of 2% α-MMP (Ofek et al., 1977). All infection assays were carried out three times in quadruplicates.

### IL-8 quantification

After 1.5 h infection (see above) the medium of the BEAS-2B cells was replaced by RPMI complete containing 100 µg/mL gentamicin and incubated for 1 h. Thereafter, the medium was replaced with RPMI complete containing 25 µg/mL gentamicin and cells were incubated O/N. The supernatant of the infected BEAS-2B cells was harvested and the release of IL-8 was determined by enzyme-linked immunosorbent assay (ELISA) as described elsewhere (Klaffenbach et al., 2011). Briefly, a 96-well plate was coated with mouse anti-human IL-8 antibody (Pierce, Rockford, USA) at 4°C O/N. The samples as well as the standard (recombinant IL-8, Peprotech, Rocky Hill, USA) were incubated for 2 h at room temperature. Bound IL-8 was detected by a mouse anti-human IL-8 biotin-coupled antibody (Pierce) followed by streptavidin-HRP (DAKO, Glostrup, Denmark). After addition of TMB substrate (BD) absorbance at 450 nm was measured using a microplate reader (Molecular Devices).

## Data availability statement

The datasets presented in this study are included in the article/**Supplementary Material** or can be found in online repositories with accession numbers provided in the article. Further inquiries can be directed to the corresponding author.

## Author contributions

RG: Conceptualization, Formal analysis, Investigation, Methodology, Supervision, Writing – original draft. IW: Writing – original draft, Formal analysis, Methodology. LH: Formal analysis, Investigation, Writing – original draft. OP: Formal analysis, Investigation, Writing – original draft. TM: Formal analysis, Investigation, Writing – original draft. LE: Formal analysis, Investigation, Methodology, Writing – original draft. CS: Formal analysis, Investigation, Writing – original draft. MH: Conceptualization, Supervision, Writing – original draft. MS: Conceptualization, Investigation, Project administration, Supervision, Writing – original draft.

## Supplementary material

Supplementary Figure 1 Effect of other antimicrobial peptides on *Salmonella* adhesion. **(A)** *S.* Typhimurium WT in LB with 5 g/L NaCl were exposed to 10 µg/mL huBPI (BPI; dark grey bar) or the BPI family member proteins BPIFA (SPLUNC1, grey bars), **(B)** BPIFB (LPLUNC1) or LBP at the concentrations indicated. Bacteria bound to tissue culture-treated plastic surfaces were quantified after incubation at 37°C for 4 h using crystal violet (CV) as described in the materials and methods section. **(C)** As a control 10 µg/mL (10, 1, 0.1) of an unrelated mouse procryptdin (procryptdin; white bars) was used in the same assay. **(D)** Biofilm formation of *S.* Typhimurium WT bacteria after 48 h incubation in LB without salt at 28°C was quantified using CV. During incubation either 5 µg/mL huBPI (dark grey bar), an equivalent amount of BPI buffer (open bar) or 0.1 µg/mL, 1 µg/mL or 10 µg/mL of human α-defensin 5 (HD5), human β-defensin 2 (HBD2) and LL-37 was present.

Supplementary Figure 2 Equal surface expression of FimH wild type and FimH T56I-containing fimbriae. **(A)** Flow cytometry analysis of surface-localized FimA of SR-11 Δ12 (solid grey), SR-11 Δ12 [p4392] (FimH WT, upper panel) or SR-11 Δ12 [FimH T56I, pWRG934] (lower panel). Expression of fimbriae from plasmids was either left uninduced (blue) or was induced with 50 ng/mL AHT (red). **(B)** Percentage of FimA-positive bacteria as gated using appropriate controls after flow cytometry analysis from three independent experiments. After induction no statistically significant (ns) difference in FimA surface expression between both plasmids was observed using a two-tailed unpaired Student’s *t* test.

Supplementary Figure 3 Mannosylation of BPI protein is important to mediate Con A binding **(A)**. Mannosylation of recombinantly produced huBPI protein or yeast mannan (YM) as indicated was tested through binding of the fluorescently labeled Man-specific lectin concanavalin A (Con A rhodamine). Mannose sensitivity of Con A rhodamine binding was tested with or without PNGase F-mediated deglycosylation of the recombinant protein. The results shown are mean ± SD of three independent experiments. Statistical significance was calculated using one-way ANOVA and was defined as ** for *p* < 0.01 and *** for *p* < 0.001. **(B)** Western blot analysis of PNGase F treated (+) or mock treated (-) rhuBPI protein.

Supplementary Figure 4 Adhesion stimulation is independent of LPS neutralizing and antimicrobial activity of huBPI. *S.* Typhimurium NCTC 12023 WT were allowed to adhere to BSA treated wells in the presence of 10 µg/mL rhuBPI (BPI) or an N-terminally truncated rhuBPI (BPI (C-term)) variant, lacking LPS neutralizing and antimicrobial activity, with or without addition of 1% α-MMP (α-MMP) as indicated. Bound bacteria were quantified after 4 h using virtual colony counts (VCC) as described in material and methods. Mean ± SD of a representative experiment out of 3 similar independent experiments done in 3- to 5-fold replicates are shown. Statistical significance was calculated using one-way ANOVA and was defined as *** for adj. *p* < 0.001.

Supplementary Table 1 Results of RNA-seq analysis.

Supplementary Table 2 Oligonucleotides used in this study.
