# Supplementary figures and images for: Subversion of a family of antimicrobial proteins by Salmonella enterica
Source: Front Cell Infect Microbiol. 2024 Mar 5;14:1375887. doi: 10.3389/fcimb.2024.1375887 (PMC10948614; doi:10.3389/fcimb.2024.1375887)

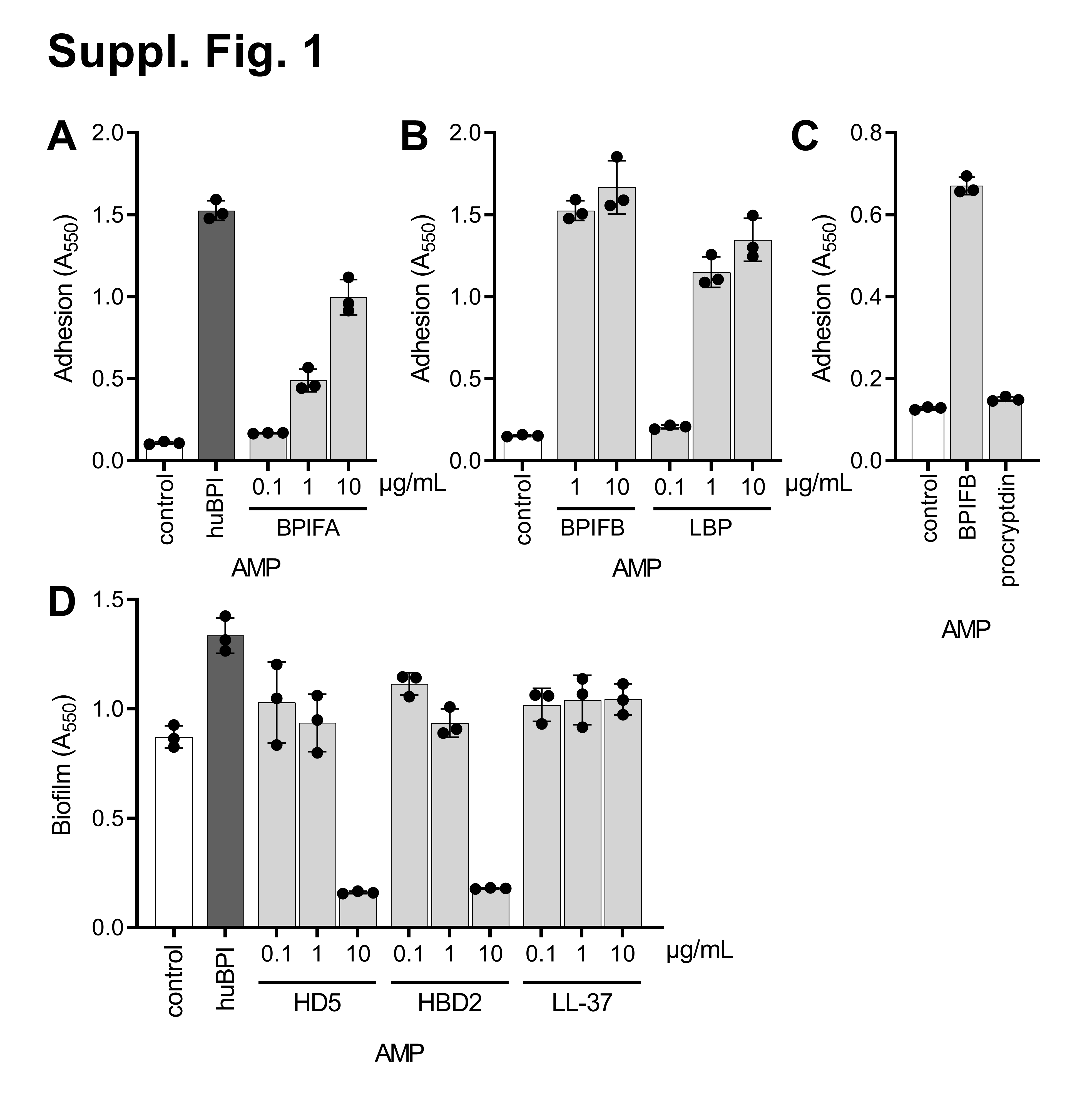

Supplement: Supplementary Figure 1 — Effect of other antimicrobial peptides on Salmonella adhesion. (A) S. Typhimurium WT in LB with 5 g/L NaCl were exposed to 10 µg/mL huBPI (BPI; dark grey bar) or the BPI family member proteins BPIFA (SPLUNC1, grey bars), (B) BPIFB (LPLUNC1) or LBP at the concentrations indicated. Bacteria bound to tissue culture-treated plastic surfaces were quantified after incubation at 37°C for 4 h using crystal violet (CV) as described in the materials and methods section. (C) As a control 10 µg/mL (10, 1, 0.1) of an unrelated mouse procryptdin (procryptdin; white bars) was used in the same assay. (D) Biofilm formation of S. Typhimurium WT bacteria after 48 h incubation in LB without salt at 28°C was quantified using CV. During incubation either 5 µg/mL huBPI (dark grey bar), an equivalent amount of BPI buffer (open bar) or 0.1 µg/mL, 1 µg/mL or 10 µg/mL of human α-defensin 5 (HD5), human β-defensin 2 (HBD2) and LL-37 was present. [file Image_1.tif]

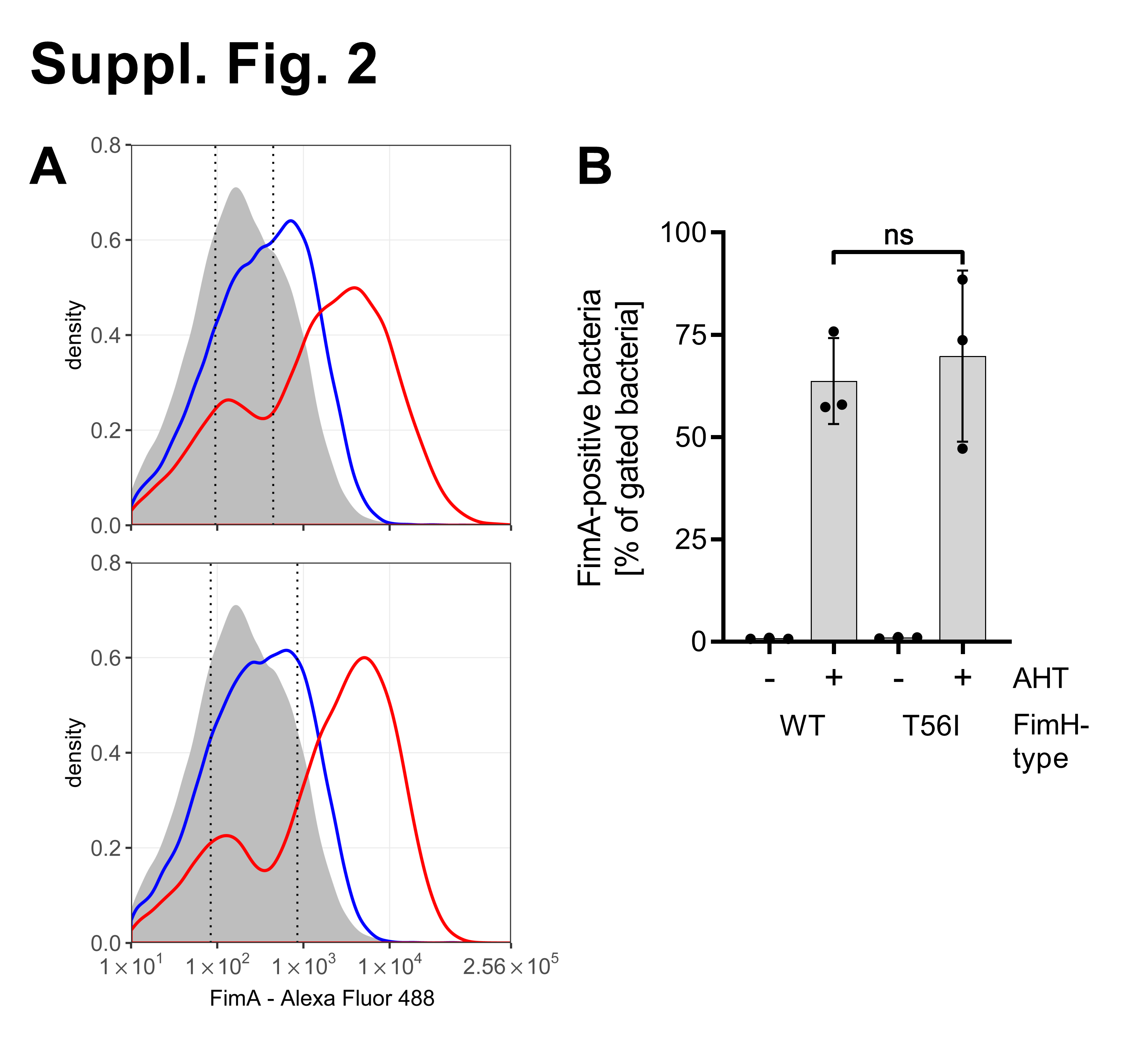

Supplement: Supplementary Figure 2 — Equal surface expression of FimH wild type and FimH T56I-containing fimbriae. (A) Flow cytometry analysis of surface-localized FimA of SR-11 Δ12 (solid grey), SR-11 Δ12 [p4392] (FimH WT, upper panel) or SR-11 Δ12 [FimH T56I, pWRG934] (lower panel). Expression of fimbriae from plasmids was either left uninduced (blue) or was induced with 50 ng/mL AHT (red). (B) Percentage of FimA-positive bacteria as gated using appropriate controls after flow cytometry analysis from three independent experiments. After induction no statistically significant (ns) difference in FimA surface expression between both plasmids was observed using a two-tailed unpaired Student’s t test. [file Image_2.tif]

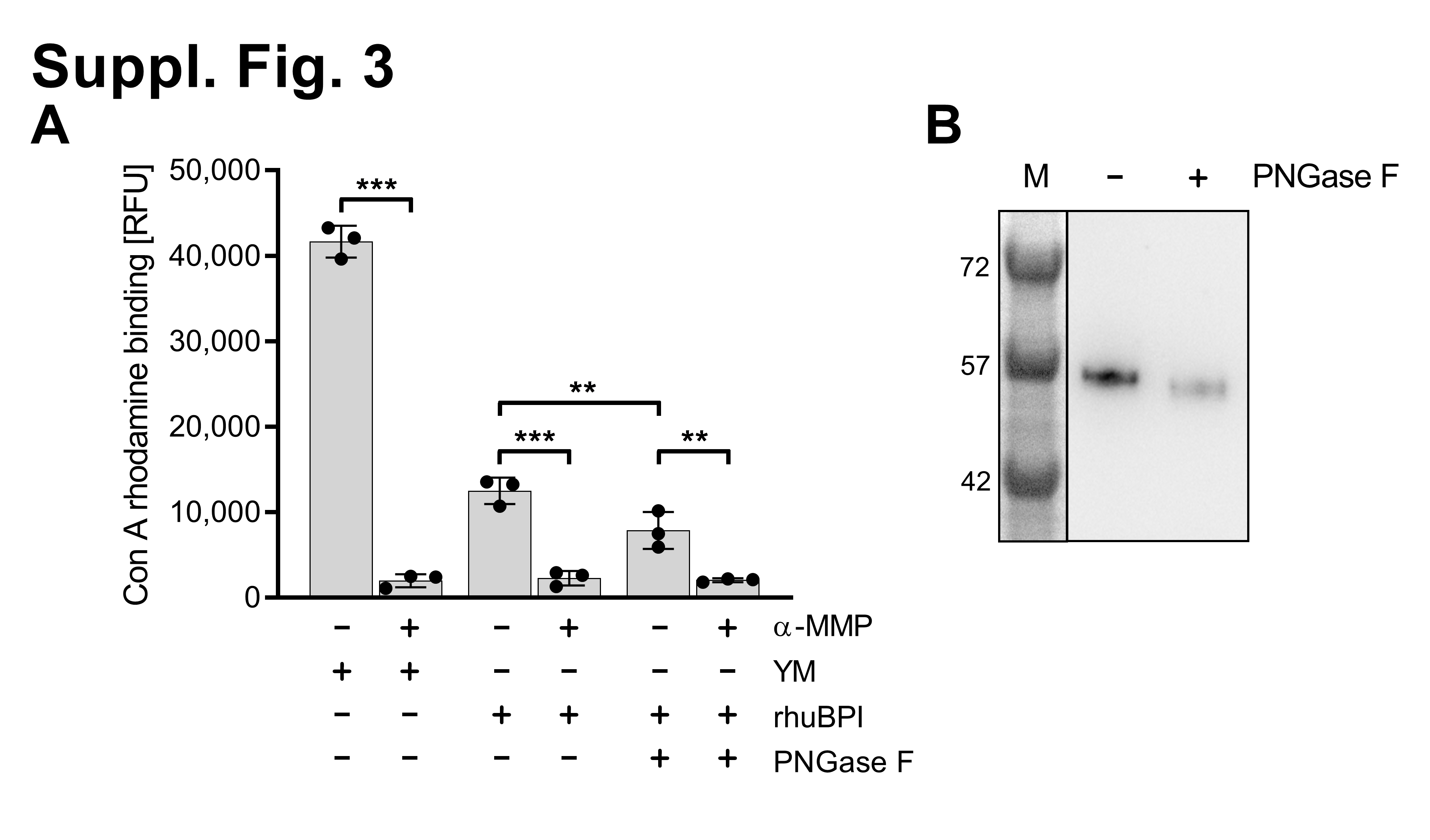

Supplement: Supplementary Figure 3 — Mannosylation of BPI protein is important to mediate Con A binding (A). Mannosylation of recombinantly produced huBPI protein or yeast mannan (YM) as indicated was tested through binding of the fluorescently labeled Man-specific lectin concanavalin A (Con A rhodamine). Mannose sensitivity of Con A rhodamine binding was tested with or without PNGase F-mediated deglycosylation of the recombinant protein. The results shown are mean ± SD of three independent experiments. Statistical significance was calculated using one-way ANOVA and was defined as ** for p < 0.01 and *** for p < 0.001. (B) Western blot analysis of PNGase F treated (+) or mock treated (-) rhuBPI protein. [file Image_3.tif]

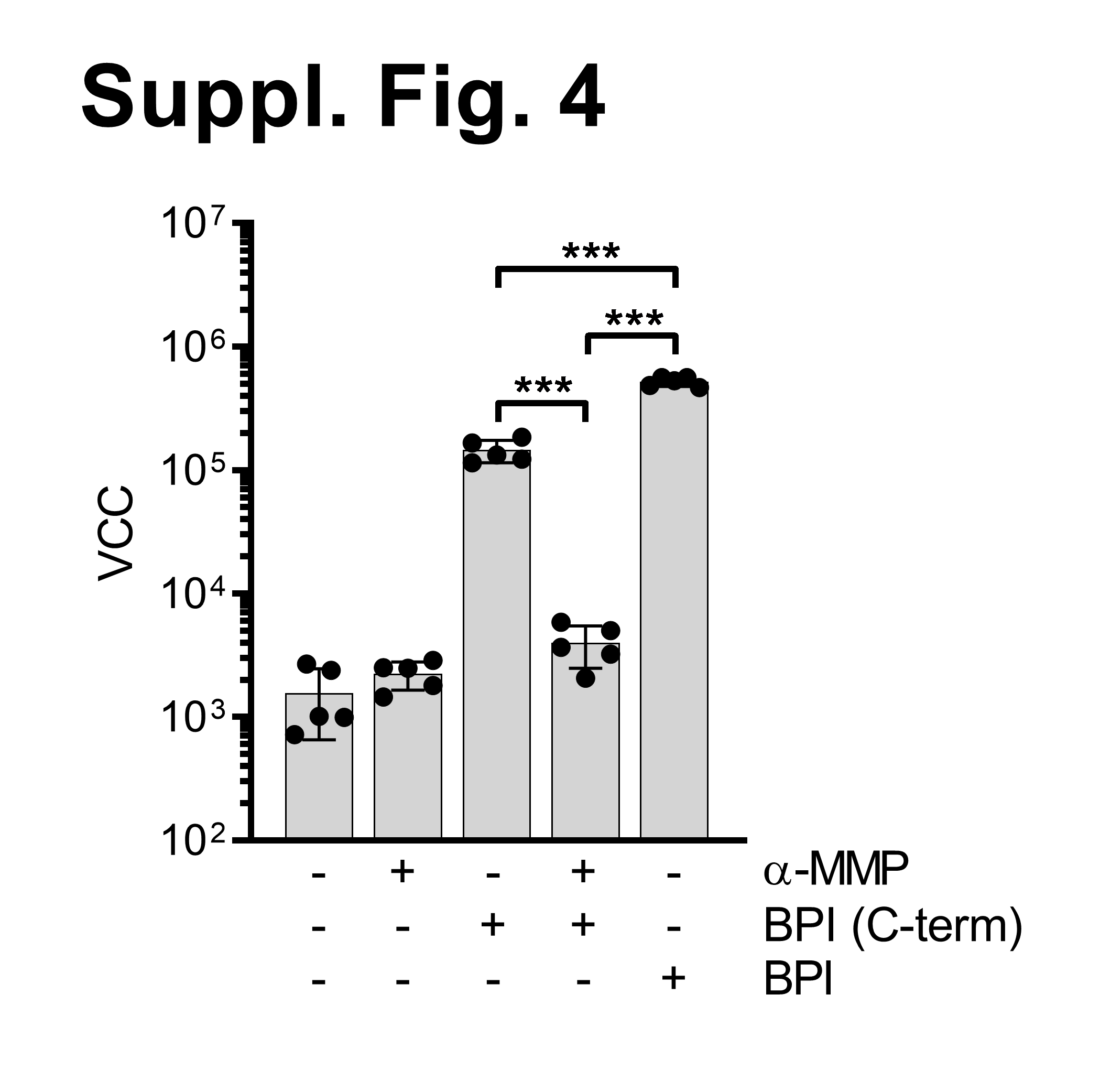

Supplement: Supplementary Figure 4 — Adhesion stimulation is independent of LPS neutralizing and antimicrobial activity of huBPI. S. Typhimurium NCTC 12023 WT were allowed to adhere to BSA treated wells in the presence of 10 µg/mL rhuBPI (BPI) or an N-terminally truncated rhuBPI (BPI (C-term)) variant, lacking LPS neutralizing and antimicrobial activity, with or without addition of 1% α-MMP (α-MMP) as indicated. Bound bacteria were quantified after 4 h using virtual colony counts (VCC) as described in material and methods. Mean ± SD of a representative experiment out of 3 similar independent experiments done in 3- to 5-fold replicates are shown. Statistical significance was calculated using one-way ANOVA and was defined as *** for adj. p < 0.001. [file Image_4.tif]
